# Cryptorchidism: Main et al. Respond

**DOI:** 10.1289/ehp.11096R

**Published:** 2008-05

**Authors:** Katharina M. Main, Jorma Toppari

**Affiliations:** University Department of Growth and Reproduction, Copenhagen, Denmark, E-mail: katharina.main@rh.regionh.dk; Departments of Physiology and Paediatrics, University of Turku, Turku, Finland

ten Tusscher and Koppe point out that maternal diabetes increases the risk of malformations in newborns and suggest that we should have excluded diabetic mothers from the data set. We agree with them that maternal diabetes increases the risk of congenital malformations, although there is only limited evidence for this in the case of cryptorchidism. In our large mother–child cohort from which this data set was derived, we found a significant association between gestational diabetes and congenital cryptorchidism (Virtanen et al. 2006).

We did not initially carry out an analysis corrected for diabetes because the biological samples were not selected with regard to diabetes; therefore data were potentially skewed for this outcome. For the same reason, the total number of mothers with diabetes in each country was low in this data set.

We have now reanalyzed the data, omitting the mothers with diabetes (2 Danish and 4 Finnish mothers for breast milk samples, and 2 Danish and 10 Finnish mothers for placentas). In a binary logistic regression analysis including all relevant confounders (maternal age, maternal prepregnancy body mass index, gestational age, weight for gestational age, parity, and country of origin), the association between the level of the seven most prevalent polybrominated biphenyl ethers (PBDEs) in breast milk (BDEs 28, 47, 66, 99, 100, 153, and 154) and congenital cryptorchidism remained significant (*p* < 0.032), with a median level (2.5th–97.5th percentiles) of 3.16 ng/g fat (1.08–21.73) in controls and 4.19 ng/g fat (1.42–52.64) in cryptorchid boys. The same analysis for the five most prevalent BDEs in placenta (BDEs 47, 153, 99, 100, and 28) remained nonsignificant (*p* = 0.173), with 1.23 ng/g fat (0.56–5.46) in controls and 1.12 ng/g fat (0.37–4.24) in cryptorchid boys.

ten Tusscher and Koppe also suggest the investigation of potential differences between mothers with and without diabetes with respect to fat content in the samples and the concentrations of PBDEs. We previously reported that the lipid content in breast milk and placenta was higher in Finnish than in Danish samples (Shen et al. 2007). Country of origin was therefore included as a covariate in a binary logistic regression. We found no significant difference between mothers with and without diabetes for fat content in breast milk or placenta (*p* = 0.975 and 0.107, respectively) or the sum of the most prevalent BDE congeners (*p* = 0.233 and 0.317, respectively). However, the number of diabetic mothers in our data set is too small to draw any firm conclusions from these results.

In conclusion, the association between perinatal exposure to PBDEs and congenital cryptorchidism was significant after exclusion of diabetic mothers. Exposure to environmental chemicals is, however, one of many adverse factors that alone, or in combination with each other, may cause testicular maldescent (Main et al. 2007). These additional factors include gestational complications, lifestyle, and genetic factors (Virtanen et al. 2007).
